# Comparison of genomic diversity and structure of sable antelope (*Hippotragus niger*) in zoos, conservation centers, and private ranches in North America

**DOI:** 10.1111/eva.12976

**Published:** 2020-04-27

**Authors:** Rebecca M. Gooley, Gaik Tamazian, Susette Castañeda‐Rico, Katherine R. Murphy, Pavel Dobrynin, Gina M. Ferrie, Holly Haefele, Jesús E. Maldonado, David E. Wildt, Budhan S. Pukazhenthi, Cody W. Edwards, Klaus‐Peter Koepfli

**Affiliations:** ^1^ Smithsonian‐Mason School of Conservation Front Royal VA USA; ^2^ Center for Species Survival, Smithsonian Conservation Biology Institute National Zoological Park Washington DC USA; ^3^ Theodosius Dobzhansky Center for Genome Bioinformatics Saint Petersburg State University St. Petersburg Russian Federation; ^4^ Center for Conservation Genomics Smithsonian Conservation Biology Institute National Zoological Park Washington DC USA; ^5^ Laboratories of Analytical Biology National Museum of Natural History Smithsonian Institution Washington DC USA; ^6^ Computer Technologies Laboratory ITMO University St. Petersburg Russian Federation; ^7^ Animals, Science and Environment Disney’s Animal Kingdom Lake Buena Vista FL USA; ^8^ Fossil Rim Wildlife Center Glen Rose TX USA; ^9^ Department of Biology George Mason University Fairfax VA USA

**Keywords:** ex situ management, genetic diversity, genetic drift, inbreeding, metapopulation, sable antelope

## Abstract

As we enter the sixth mass extinction, many species that are no longer self‐sustaining in their natural habitat will require ex situ management. Zoos have finite resources for ex situ management, and there is a need for holistic conservation programs between the public and private sector. Ex situ populations of sable antelope, *Hippotragus niger*, have existed in zoos and privately owned ranches in North America since the 1910s. Unknown founder representation and relatedness has made the genetic management of this species challenging within zoos, while populations on privately owned ranches are managed independently and retain minimal‐to‐no pedigree history. Consequences of such challenges include an increased risk of inbreeding and a loss of genetic diversity. Here, we developed and applied a customized targeted sequence capture panel based on 5,000 genomewide single‐nucleotide polymorphisms to investigate the genomic diversity present in these uniquely managed populations. We genotyped 111 sable antelope: 23 from zoos, 43 from a single conservation center, and 45 from ranches. We found significantly higher genetic diversity and significantly lower inbreeding in herds housed in zoos and conservation centers, when compared to those in privately owned ranches, likely due to genetic‐based breeding recommendations implemented in the former populations. Genetic clustering was strong among all three populations, possibly as a result of genetic drift. We propose that the North American ex situ population of sable antelope would benefit from a metapopulation management system, to halt genetic drift, reduce the occurrence of inbreeding, and enable sustainable population sizes to be managed ex situ.

## 1 INTRODUCTION

Ex situ conservation breeding mitigates the probability of extinction for many endangered species that are declining or no longer self‐sustaining in their natural habitat (Conde, Flesness, Colchero, Jones, & Scheuerlein, 2011). The goal of ex situ conservation breeding is to increase the number of individuals to ensure a stable population and manage genetic diversity through selective breeding and inbreeding avoidance (Hoffmann, Sgro, & Kristensen, 2017). Various factors including founder effects, selection, random genetic drift, and inbreeding can alter the fitness and genetic diversity of a population (Frankham, 2008;Gooley, Hogg, Belov, & Grueber, 2017;Lacy, 1987;Wright, 1931), and in the absence of appropriate management, such processes can result in adaptation to captivity, genetic erosion, and inbreeding depression (Frankham, Ballou, & Briscoe, 2002;Jule, Leaver, & Lea, 2008;Williams & Hoffman, 2009). These processes have been identified as possible contributors to declines in reproductive fitness and neonatal survival in captivity as well as the historically limited success of species reintroductions (Christie, Marine, French, & Blouin, 2012;Farquharson, Hogg, & Grueber, 2018;Fischer & Lindenmayer, 2000;Willoughby & Christie, 2019). Gaining an understanding of genetic diversity within ex situ populations and providing best practice guidelines to retain this diversity is common among breeding programs (e.g., Gautschi, Muller, Schmid, & Shykoff, 2003;McLennan et al., 2018).

The World Association of Zoos and Aquariums (WAZA) provides global guidelines to help manage species in captivity, some of which aim to combat the above‐mentioned challenges through the maintenance of genetic health and sufficient population sizes (WAZA, 2005). Mean kinship (MK) pairing is the most widely used genetic management strategy within zoo populations. Using pedigree‐derived MK values (the average kinship of an individual to the entire captive population, including itself), the least related and under‐represented individuals are prioritized and recommended for breeding (Ballou & Lacy, 1995). Minimizing the MK of each generation in ex situ populations, in theory, should retain greater genetic diversity when compared to random mating (Ballou & Lacy, 1995), as each individual contributes equally to the population. However, while minimizing MK has been shown to effectively retain founder genetic diversity in both laboratory and simulated populations (Ivy & Lacy, 2012;Willoughby et al., 2015), it can be challenging to implement such a strategy in certain ex situ management conditions. For example, in group housing enclosures where the pedigree of offspring is unknown (e.g., Farquharson, Hogg, & Grueber, 2019;Gooley, Hogg, Belov, & Grueber, 2018) or in polygamous species such as antelope, it may be difficult to equalize male reproductive success (e.g., Mucha & Komen, 2016).

Additionally, maintaining sufficient population sizes ex situ, especially for large animals that require spacious enclosures, can be challenging in a traditional urban zoo setting (Wildt et al., 2019). For example, ungulates managed in accredited Association of Zoos and Aquariums (AZA) zoos rarely meet sustainability goals, with only one species program considered genetically sustainable (projected to retain 90% of the founding genetic diversity for 100 years) and only 31.5% of managed ungulate species having a population size greater than 100 (Association of Zoos & Aquariums, 2019). The Source Population Alliance (SPA; www.sourcepopulation.org, Wildt et al., 2019), a program implemented through the Conservation Center for Species Survival (C2S2; www.conservationcenters.org), aims to tackle this challenge by bringing together urban zoos, conservation centers, and privately owned ranches to manage select species collectively as metapopulations. The SPA’s initial focus is on four ungulate species (the scimitar‐horned oryx, *Oryx dammah*; the dama gazelle, *Nanger dama*; the addax, *Addax nasomaculatus;* and the sable antelope, *Hippotragus niger*) that are not only of high conservation value, but also have large population sizes in the North American private sector relative to the number of animals in the AZA’s Species Survival Plan (SSP^®^) programs for these species (Wildt et al., 2019).

The SPA brings together institutions that have varying degrees of genetic management, ranging from AZA‐accredited zoos implementing pedigree‐based breeding recommendations, to privately owned ranches of isolated, nonpedigreed populations. However, even within AZA populations, solely relying on pedigree‐based genetic management has been difficult for ungulates (see for example the sable antelope SSP^®^ [Piltz, Sorensen, & Ferrie, 2015], the addra gazelle (*N. d. ruficollis*) SSP^®^ [Thier & Spevak, 2017], the Père David's deer (*Elaphurus davidianus*) SSP^®^ [Schille & Ferrie, 2016], the Barasingha swamp deer (*Rucervus duvaucelii*) SSP^®^ [Gelvin & Nuetzel, 2019], and the river hippopotamus (*Hippopotamus amphibius*) SSP^®^ [Davis & Lynch, 2018]), as unknown founder relations and incomplete pedigrees have compromised the ability of managers to provide breeding recommendations that effectively retain genetic diversity. In fact, of 31 ungulate species with managed SSP^®^ populations, 26 species have some percentage of unknown parentage and are managed based on assumptions of relatedness (R.M. Gooley *unpublished data*). Integrating privately owned populations into pedigree‐based breeding management plans requires an understanding of kinship of individuals and the genetic diversity present in historically isolated populations to enable a metapopulation management approach.

In this study, we compared genetic diversity in southern sable antelope (*H. n. niger)* populations located in zoos, conservation centers, and private ranches in North America that have been managed using different breeding protocols, to understand the genetic differentiation that has occurred in North American animals since their founding. Specifically, we analyzed genetic diversity using single‐nucleotide polymorphism (SNP) data, obtained from a sable antelope‐specific targeted sequence capture SNP panel (designed herein). The SNPs selected for this analysis were derived from previously reported sable antelope whole genome sequences (Koepfli et al., 2019). Our study aimed to (a) quantify the genetic diversity and structure present in AZA zoo populations, conservation centers, and privately managed ranch populations in North America and make inferences between captive management strategies and retention of genetic diversity and, (b) for the first time, provide management teams with new genomic information to make better breeding recommendations for ex situ populations of sable antelopes.

## 2 MATERIALS AND METHODS

### 2.1 Study species and sample acquisition

The sable antelope consists of five genetically distinct and geographically coherent populations distributed across eastern and southern Africa in woodland savanna habitat (Vaz Pinto, 2018, 2019). Sable antelope are gregarious, forming herds consisting of multiple females and a single breeding male. Bachelor herds are comprised of juvenile and nonbreeding males (Vaz Pinto, 2019). Of the five populations, four of which have been recognized as subspecies, the giant sable (*H. n. variani*) is listed as critically endangered in The IUCN Red List of Threatened Species, and the remaining three subspecies (the Zambian sable, *H. n. kirkii*; the southern sable, *H. n. niger*; and the eastern sable, *H. n. roosevelti*) are listed as least concern (IUCN/SSC Antelope Specialist Group, 2017). However, recent population assessments indicate their numbers in the wild may be declining, as the species has lost at least 51% of its former range (Ripple et al., 2015) and once formerly contiguous populations have become fragmented and isolated.

The current estimated population of sable antelope in the wild is between 50,000 and 60,000 (IUCN/SSC Antelope Specialist Group, 2017). For the southern sable antelope subspecies, populations across South Africa, Zimbabwe, Mozambique, Botswana, and Namibia totaled ~37,000 animals, based on estimates collected in the late 1990s (East, 1999). In the North American public sector, including both accredited AZA zoos and conservation centers and some non‐AZA zoos, the ex situ population numbers 129 individuals (38 males, 90 females, 1 unknown) located in 15 institutions, with herd sizes ranging from 3 to 39 animals (Piltz et al., 2015). The private ex situ population in North America, however, is estimated to be over 3,000 (Mungall, 2018). These North American populations are almost entirely comprised of animals representing the southern sable antelope subspecies.

Presently, only 35% of the pedigree is considered to be known for the AZA managed population of sable antelope, with several parental assumptions (hypothetical parents to group family lineages) and exclusions (individuals which are removed due to sterility or age) (Piltz et al., 2015). This decreases to 27% prior to parental assumptions. Including pedigree assumptions, 39 founders have contributed to the AZA population, but their original relatedness and their representation is largely unknown. As the majority of the pedigree remains incomplete, MK values are unable to be calculated; as a result, current breeding recommendations achieve inbreeding avoidance via breeding males being rotated among AZA facilities every two to three years.

For the purpose of this study, three urban zoo AZA facilities were analyzed together as a population referred to as “AZA” (details about origins and sample sizes are summarized in Table 1). The “AZA” population is intensively managed with small herds or breeding pairs. Sable antelope from Fossil Rim Wildlife Center (an AZA‐accredited facility) were analyzed as a separate population, due to the considerably larger herd size and less intensive management relative to urban AZA zoos. Samples were also obtained from four privately owned sable antelope populations that are participants in the Source Population Alliance (SPA). For the purpose of this study, all four ranch populations were analyzed together as a population referred to as “Ranch.”

**Table 1:** Sources of sable antelope samples used in this study with regard to institution, sector, management strategy, and enclosure size

Institution	Sector	*n* ^a^	Herd size	Management and enclosure size
Disney's Animal Kingdom^®^	AZA	8	12	Intensive/urban^b^
Omaha's Henry Doorly Zoo and Aquarium	AZA	3	16	Intensive/urban
The Wilds	AZA	12	9	Less intensive^c^
Fossil Rim Wildlife Center	AZA	43	45	Less intensive
Ranch A	Private	1	30	Least intensive/vast^d^
Ranch B	Private	5	20	Least intensive/vast
Ranch C	Private	11	30	Least intensive/vast
Ranch D	Private	28	28	Least intensive/vast

^a^ Samples were collected over several years, during which time herds transferred and received individuals.

^b^ Genetically managed breeding pairs or small breeding groups, in urban zoo enclosures.

^c^ Genetically managed breeding groups or herds, in large enclosures.

^d^ Nonmanaged breeding herds, in large enclosures.

Sample selection within the AZA managed population (including Fossil Rim) was guided using the software program PedSam (https://people.uwm.edu/latch/software‐2/), in an effort to select individuals with unresolved pedigrees and to have all assumed founding lineages represented in our study. PedSam requires a full analytical pedigree (complete from founding generation) and uses current status (living, lost‐to‐follow‐up, or deceased), known relations and unresolved parentage to select a subset of informative individuals for sampling, from living individuals and individuals that have already been sampled. From our PedSam analysis, 89 individuals were required for sampling in order to resolve the AZA sable antelope pedigree and/or obtain a genetically representative sample set of the current living population. Samples were available for 42 of the required individuals and were subsequently used for genotyping in this study. In addition, 24 individuals that were not on our required sampling list were also genotyped, as samples were readily available (see Table 1 for sample distribution across AZA facilities).

In total, whole blood samples (1–4 ml) or skin biopsies via biopsy darts were acquired from 111 sable antelope (66 from four AZA zoos and 45 from four privately owned ranches). All samples were collected opportunistically in conjunction with routine neonate examination/ear tagging/medical procedures. Therefore, no animal care and use approval was required by the Smithsonian Institution or the various collaborating institutions.

### 2.2 Design of the sable antelope targeted sequence capture SNP panel

We used the SNPs previously identified from a reference southern sable antelope genome assembly and the re‐sequenced whole genomes of four southern and one Zambian sable antelope derived from ex situ populations (Koepfli et al., 2019) to custom design a sequence capture panel containing 5,000 SNPs distributed across the sable antelope genome. Putatively neutral SNPs were selected from nonrepetitive (repeat‐masked) and noncoding (outside exons based on gene annotation) regions and filtered according to the GC content skew. SNPs containing gaps in their flanking sequences (100 bp upstream and downstream) were excluded. To minimize linkage, we selected SNPs that were located at least 10 kbp apart in the scaffolds of the sable antelope genome assembly reported in Koepfli et al. (2019). Using these filtering criteria, we randomly selected 5,000 SNPs distributed across the genome.

To visualize the distribution pattern of the 5,000 selected SNPs across each of the 29 autosomes and X chromosome of the sable antelope genome, we mapped the SNP positions from the 16,927 scaffolds of the sable antelope genome assembly reported in (Koepfli et al., 2019) to scaffolds of the sable antelope chromosome‐length assembly downloaded from the DNA Zoo (https://www.dnazoo.org/assemblies/Hippotragus_niger) using a custom Python script. We then visualized the density and distribution of SNPs across the 30 largest chromosome‐length scaffolds by splitting the scaffolds into 100 kbp nonoverlapping windows and highlighting the windows with the selected SNPs.

### 2.3 Construction of the sable antelope SNP panel

The 5,000 SNPs and their associated flanking regions were submitted to Arbor Biosciences for myBaits^®^ screening and design. Each SNP (bait) candidate was first BLASTed against the sable antelope scaffolded genome (Koepfli et al., 2019). For each BLAST hit, a melting temperature (Tm) of hybridization was predicted using salt and temperature conditions matching the bait hybridization conditions. Then, for each bait candidate, the number of BLAST hits with Tms falling into the following six temperature bins were counted: 40–60°C, 60–62.5°C, 62.5–65°C, 65–67.5°C, 67.5–70°C, and above 70°C. We note that during bait sequence capture, the hybridization temperature is 65°C and, by definition, Tm represents the temperature where 50% of the molecules are hybridized to their complementary strand. Each of the 5,000 SNP/bait candidates included a final flanking sequence length of 60 bp on either side of the SNP so that each locus consisted of 120 bp. The final myBaits^®^ sequence capture contained 20,000 probes with a ~4x tiling density (~15 bp probe spacing).

### 2.4 Genomic DNA isolation

For each individual in our study, genomic DNA was extracted from either 100 or 300 μl of whole blood or a skin biopsy punch. For blood samples, DNA was extracted using either the DNeasy Blood and Tissue Kit (Qiagen) or FlexiGene DNA kit (Qiagen) following the manufacturer's protocol and eluted in 200 μl 10 mM Tris, pH 8.0. Genomic DNA was extracted from biopsy punches using the GENE PREP system (AutoGen) and eluted in 100 μl of R9 buffer (AutoGen). Following extraction, DNA samples were quantified by dsDNA High‐Sensitivity (HS) Qubit (Invitrogen).

### 2.5 Library preparation and capture enrichment

As sable antelope samples used for genotyping were collected over several years, genomic libraries for targeted sequence capture were prepared in two separate batches using two different protocols (1 and 2) in two laboratories in different years (2017 and 2019). For protocol 1, 100 μl of each genomic DNA extraction was normalized to 500 ng/μl and sheared to an average of ca. 500 bp using a Q‐Sonica Q800R3 sonicator (Qsonica) with an amplitude of 25%, a pulse of 15:15, and a temperature of 4°C. Postshearing, samples were cleaned and concentrated using 2x solid‐phase reversible immobilization (SPRI) magnetic beads (Beckman Coulter) to remove small fragments then eluted in 22 μl of double‐distilled water.

Each sample was prepared as a dual‐indexed library using the KAPA LTP Library Preparation Kit for Illumina^®^ Platforms sequencing following the manufacturer's protocol (KAPA Biosystems, version 6.17), with one quarter reaction volumes. Dual‐indexing PCR was performed with Nextera‐style indices (Faircloth & Glenn, 2012) using KAPA HiFi Hotstart Ready Mix (KAPA Biosystems) according to the manufacturer's protocols, with an initial denaturation of 98°C for 2 min followed by 14 cycles of 98°C for 30 s, 65°C for 30 s, 72°C for 60 s, and a final extension of 72°C for 10 min. The indexed libraries were purified using 1.6x SPRI magnetic beads (Beckman Coulter; DeAngelis, Wang, & Hawkins, 1995). Library concentrations were quantified using the Qubit^®^ DNA High Sensitivity Kit, and library size ranges and qualities were inspected using a Bioanalyzer 2,100 with High Sensitivity DNA kits (Agilent Technologies). Libraries were pooled equimolarly in quartets. SNPs were then enriched using the sable antelope myBaits^®^ Custom Target Capture Kit (Arbor Biosciences) following the myBaits protocol version 3. Postenrichment libraries were amplified using KAPA HiFi Hotstart Ready Mix (KAPA Biosystems) with an initial temperature of 98°C for 2 min followed by 14 cycles of 98°C for 20 s, 60°C for 30 s, 72°C for 45 s, and a final extension of 72°C for 5 min, purified using 1.6x SPRI magnetic beads, and quantified by Qubit^®^ DNA High Sensitivity Kit. Size ranges and qualities of libraries were inspected using a Bioanalyzer 2,100 with High Sensitivity DNA kits (Agilent Technologies) and pooled in equimolar ratios.

For protocol 2, genomic DNA samples were sheared to an average size of approximately 200 bp using a Covaris ME‐220 Focused Ultrasonicator with the following settings: 23 iterations; 10 s repeat process treatment duration; 50W peak incident power; 30% duty factor; 1,000 cycles per burst; and 12°C temperature. The fragmented DNA was then purified using a 1.5X bead clean with KAPA Pure beads (KAPA Biosystems). For target enrichment, 10 ng of purified, fragmented DNA was used to construct a whole genome sequencing (WGS) library for each sample using the KAPA Hyper Prep Library Kit with amplification and dual combinatorial indexing (KAPA Biosystems). Each library was then purified with a 1X bead clean with KAPA Pure beads (KAPA Biosystems).

Following library construction, samples were target‐enriched from 100 ng of each WGS library using the sable antelope myBaits^®^ Custom Target Capture Kit (Arbor Biosciences) per the manufacturer's protocol. The enriched libraries were then re‐amplified with KAPA HiFi HotStart ReadyMix (KAPA Biosystems) using the universal reamplification primers described in Meyer and Kircher (2010) with the following thermocycler conditions: 98°C for 2 min; 14 cycles of 98°C for 20 s, 60°C for 30 s, 72°C for 30 s; and a final extension at 72°C for 5 min. Lastly, the enriched libraries were purified using a 1X bead clean with KAPA Pure Beads (KAPA Biosystems) and eluted in 17 μl of 10 mM Tris, pH 8.0. The target‐enriched libraries were then quantified by dsDNA HS Qubit (Invitrogen), sized on a TapeStation 2200 (Agilent Technologies), and pooled in equimolar ratios. The final pool was quantified by qPCR using a KAPA library quantification kit (KAPA Biosystems). For both protocols, negative controls were used to account for contamination. The pooled libraries generated by protocols 1 and 2 were each submitted to the Psomagen Clinical Laboratory and paired‐end sequenced (2 × 150 bp) on one lane of an Illumina HiSeq X Ten instrument.

### 2.6 Variant calling

Sequencing reads from 111 sable antelope were aligned to the sable antelope reference genome (Koepfli et al., 2019) using the Burrows–Wheeler Aligner (BWA; version 0.7.17 [Li & Durbin, 2009]). SAMtools fixmate (version 1.6 [Li et al., 2009]) was performed initially to ensure mate‐pair information was concordant between paired‐end reads for downstream variant calling. As insertions and deletions (indels) may produce false SNP calls as a result of misalignment, we performed a local realignment around indels using GATK IndelRealigner (version 3.7 [DePristo et al., 2011]). Read groups were added to each sample using the Picard tools readgroup command (version 2.5.0 [http://broadinstitute.github.io/picard/]). Duplicates, which can arise during library preparation, were marked using Picard tools MarkDuplicates (version 2.5.0 [http://broadinstitute.github.io/picard/]) and masked from downstream analyses. Variants were called using bcftools mpileup (version 1.4.1 [Li et al., 2009]). Variant calls were performed within the genomic coordinates of the designed SNP sequence capture panel, to prevent off target variant calling. The final output VCF file was filtered for variant quality (quality minimum of 20), minor allele count (count minimum of 10), and minimum read depth (depth minimum of 10) using VCFtools (version 0.1.14 [Danecek et al., 2011]). VCFtools was also used to remove indels from downstream analyses (Danecek et al., 2011). Using PLINK (version 1.9 [Purcell et al., 2007, http://pngu.mgh.harvard.edu/purcell/plink/ ]), our VCF file was transformed into a.ped PLINK format file and filtered for variants with a minor allele frequency below 0.05, variants with missing call rates exceeding 0.05 and pruned for variant pairs in linkage disequilibrium (LD) with an *R* ^2^ correlation coefficient of 0.5 or greater (4,037 SNPs retained after filtering). Finally, we used PLINK to filter any SNPs deviating from Hardy–Weinberg equilibrium, with significance set to *p* < .001. After filtering, all 111 sable antelope remained and 3,954 of the initial 5,000 SNPs were retained, which were used for all downstream population genetic analyses.

### 2.7 Genetic diversity analyses

Individual observed heterozygosity was calculated using PLINK (Purcell et al., 2007, http://pngu.mgh.harvard.edu/purcell/plink/). We performed Wilcoxon–Mann–Whitney tests comparing population heterozygosity between our three North American populations (AZA zoos, Fossil Rim, and Ranches). Statistical significance was set at *p* < .05.

Realized inbreeding coefficients for each individual were calculated in R (R Core Team, 2019) using the RZooRoH package (Bertrand et al., 2019). This package implements a hidden Markov model that uses the observed succession of individual SNP genotypes to categorize individual genomes into segments of homozygosity‐by‐descent (HBD, evident from runs of homozygosity; RoH) segments and non‐HBD segments (Bertrand et al., 2019). As RoH and the lengths of such runs are informative of past inbreeding events, RZooRoH categorizes the lengths of HBD segments into generation classes. Longer RoHs correspond to more recent inbreeding events (fewer generations past) and shorter RoHs correspond to more ancestral inbreeding events (many generations past). As the low density of our filtered SNP panel limits our ability to detect shorter HBD segments associated with more ancestral inbreeding events, we set our model to eight HBD classes (*k*) and one non‐HBD class using a predefined *zoomodel* (with *R_k_* equal to 2, 4, 6, 8, 10, 12, 14, and 16 for HBD and 16 for non‐HBD). *R_k_* values are approximately double the number of generations from the time of inbreeding, and we can therefore track inbreeding events that have occurred since the establishment of the captive sable population in North America approximately 8.4 generations ago (based on the IUCN sable generation length of 7.1 years [IUCN/SSC Antelope Specialist Group, 2017]). The output provides both the overall realized inbreeding coefficient for each individual and estimates the contribution of each predefined HBD class to the overall inbreeding coefficient. We performed Wilcoxon–Mann–Whitney tests comparing population realized inbreeding between our three North American populations (AZA zoos, Fossil Rim and Ranch). Statistical significance was set at *p* < .05.

### 2.8 Genetic structure analyses

To detect genetic clustering among the three designated North American populations, we performed a principle component analyses (PCA) using the R package SNPRelate (Zheng et al., 2012). Coordinates for principle component 1 and principle component 2 were plotted using the R package *ggplot2* (Wickham, 2009).

## 3 RESULTS

### 3.1 Distribution of SNPs across the sable antelope genome

**Figure 1:**
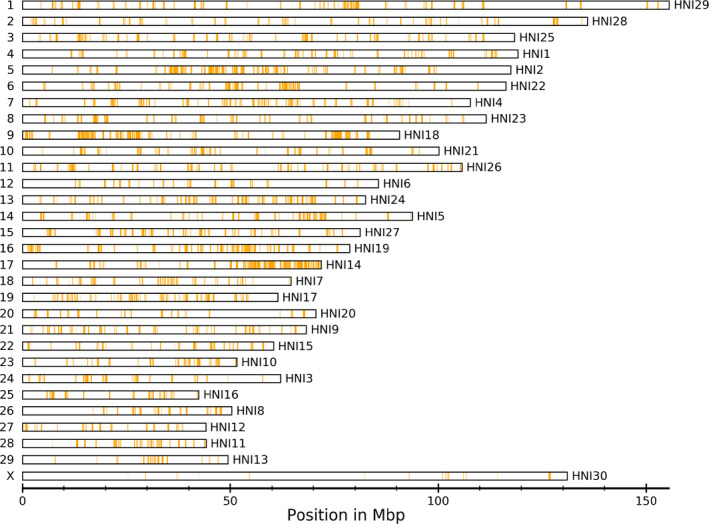
The chromosomal distribution of 4,353 (out of a total of 5,000) SNPs selected for the design of the sable antelope sequence capture panel. SNPs are shown as orange bars. The chromosome‐length scaffolds are ordered from top to bottom according to their alignment to the domestic cow chromosome map (UMD3.1; Zimin et al., 2009), which was performed using LAST (Kiełbasa, Wan, Sato, Horton, & Frith, 2011). Numbers shown on the left of scaffolds are chromosome numbers or sex chromosomes of the domestic cow, while numbers shown on the right are the corresponding scaffolds of the sable antelope genome assembly (see: https://www.dnazoo.org/assemblies/Hippotragus_niger). Only the 30 chromosome‐length scaffolds are shown. Length of scaffolds in Mbp (x‐axis)

Out of the 5,000 SNPs selected for the design of the myBaits^®^ sequence capture panel, 4,353 SNPs mapped to the 30 chromosome‐length scaffolds of the sable antelope assembly (https://www.dnazoo.org/assemblies/Hippotragus_niger). Figure 1 shows the distribution of these SNPs across the 30 chromosome‐length scaffolds of the sable antelope genome, ordered according to the domestic cow chromosome map. The cow X chromosome corresponds to scaffold 30 in the sable antelope chromosome‐length assembly, which contained 27 SNPs. The remaining 647 SNPs were located on smaller, unplaced scaffolds (not shown). A frequency distribution of the distances between the 4,353 SNPs on the 30 chromosome‐length scaffolds showed a median distance of 43,314 bp, range = 396–10,757,538 bp, and 5%‐95% percentile range = 12,387–227,434 bp (Figure S1).

### 3.2 Alignment rate of targeted sequence capture reads

We analyzed the aligned reads using the samtools flagstat tool from the SAMtools package (Li et al., 2009). The alignment rate of reads generated from the two targeted sequence capture protocols (1 and 2) to the sable antelope genome had a mean value of 98.8% (4.2 million reads) across the 111 sable antelope samples (Figure S2). We detected no batch effects as the two protocols had a similar mean rate of read alignment (98.4% for protocol 1% versus 99.1% for protocol 2). However, one sample was an outlier, with only 47.9% (1.3 million) reads aligned. Despite this low alignment rate, inspection of the resulting VCF file of this sample suggested no aberrant genotype calls, and therefore, it was included in the population genetic analyses.

### 3.3 Genetic diversity analyses

**Figure 2:**
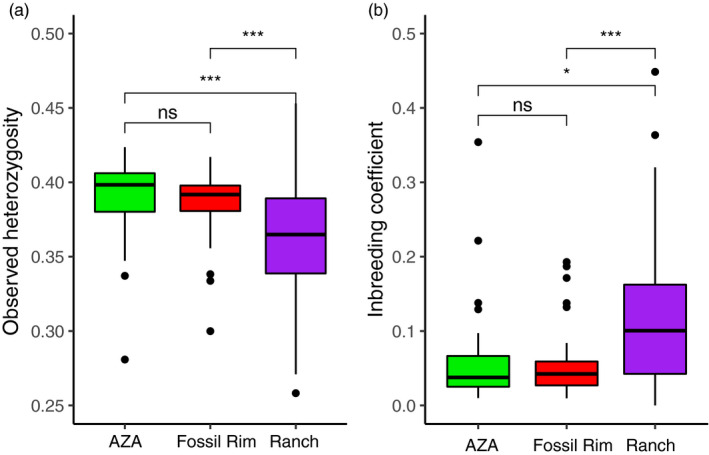
Genetic diversity calculated from 3,954 SNPs in sable antelope from three North American ex situ populations. (A) Observed heterozygosity from the three populations; both AZA zoos and Fossil Rim have significantly higher heterozygosity than the Ranch populations. (B) Realized inbreeding coefficients from the three populations; both AZA zoos and Fossil Rim have significantly lower inbreeding coefficients than the Ranch populations. Significance was calculated performing a Wilcoxon–Mann–Whitney test: ns, not significant, *significance at *p* < .05, **significance at *p* < .01, ***significant at *p* < .005

After filtering, 3,954 SNPs and all 111 sable antelope samples remained for analysis. Individual observed heterozygosity ranged from 0.23 to 0.45 (mean 0.368 *SD* ± 0.04; Figure 2a). We found no significant difference in observed heterozygosity between the AZA zoos and Fossil Rim. Observed heterozygosity of sable antelope from the Ranch populations had a larger range in heterozygosity than both AZA and Fossil Rim and had significantly lower populationwide heterozygosity than both AZA (*p* < .01) and Fossil Rim (*p* < .01; Wilcoxon–Mann–Whitney test; Figure 2a).

**Figure 3:**
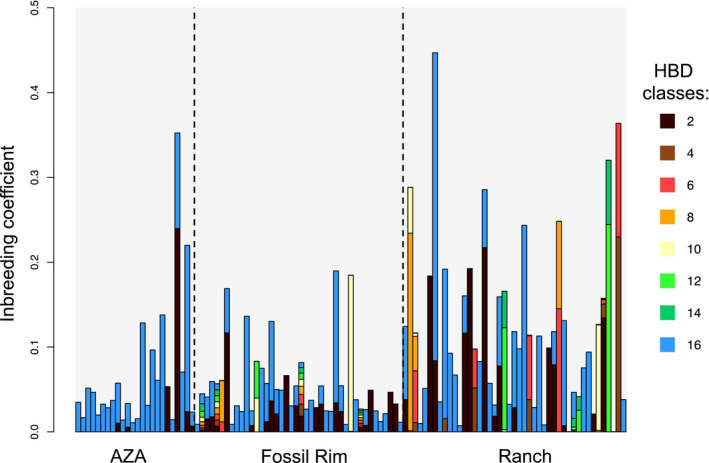
Realized inbreeding coefficients calculated from 3,954 SNPs using the RZooRoH model in three North American populations of sable antelope. Each bar represents an individual, displaying overall individual inbreeding coefficients (y‐axis) and the proportion of genome assigned to specific homozygosity by descent (HBD) classes. HDB class values are assigned in concordance with the length of the run of homozygosity, where longer runs of homozygosity indicate more recent inbreeding events. The color legend indicates the HBD class, with class numerical values corresponding to approximating double the generation value of an inbreeding event (e.g., class 2 [color black] corresponds to inbreeding events occurring between ancestors 1 generation past, and class 16 [color blue] corresponds to inbreeding events occurring between ancestors 8 generations past). See Materials and Methods section 2.8 for further details

Individual realized inbreeding coefficients ranged from 0.00 to 0.45 (0.089 ± 0.08, mean ± *SD*; Figure 2b & Figure 3). We found no significant difference between AZA zoos and Fossil Rim population‐wide inbreeding coefficients. Ranch inbreeding coefficients had a larger range and were significantly higher than those from both AZA zoos and Fossil Rim (Wilcoxon–Mann–Whitney test *p* < .05 and *p* < .005; Figure 2b). The Ranch population appeared to have experienced more recent inbreeding events (black bars; indicating ancestors interbred approximately one generation ago), more frequently than sable antelope in AZA zoos (Figure 3), and in general showed a wider mosaic of ancestral inbreeding events. See, for example, the second individual within the Ranch population in Figure 3, which displays inbreeding events from ancestors interbreeding approximately four and five generations ago (orange and yellow segments). This is in direct contrast to the AZA population, where the majority of individuals show inbreeding events from ancestors interbreeding eight generations past (blue segments; pre‐AZA management) and very minimal recent inbreeding events.

### 3.4 Genetic structure analyses

**Figure 4:**
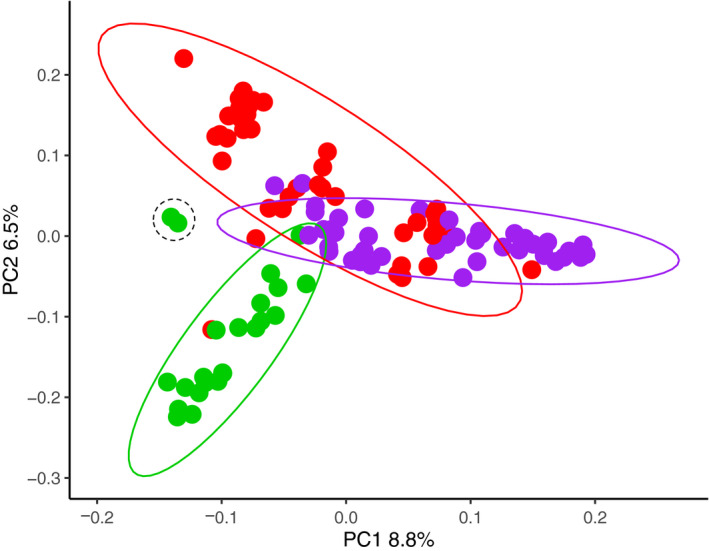
Principal component analysis calculated from 3,954 SNPs for three sable antelope populations located in North America (AZA accredited zoo population = green, AZA accredited conservation center [Fossil Rim] = red, privately managed ranch population = purple); PC1 explains 8.8% of the observed variation, and PC2 explains 6.5% of the observed variation. Populations show moderate to distinct clustering as indicated by colored ellipses. Two offspring, sired from a Fossil Rim individual transferred to an AZA accredited zoo, are indicated in the dashed black colored ellipse

Our principal component analysis (PCA) comparing North American sable antelope populations showed that each population formed an obvious cluster (Figure 4). However, sable antelope that originated from Texas (Fossil Rim and the Ranch populations) showed overlapping clusters. Sable antelope from AZA zoos comprised a largely distinct cluster along both axes of the plot.

## 4 DISCUSSION

This study is the first to compare genetic diversity, genetic clustering, and genetic drift among a subset of sable antelope managed in zoos, conservation centers, and ranches, and to use this information to propose and support metapopulation management between traditionally isolated populations. Using a customized SNP panel developed within this study, our findings indicate that captive sable antelope populations managed by the Association of Zoos and Aquariums (AZA) retain significantly greater genetic diversity and have significantly lower inbreeding coefficients than populations managed in the privately owned ranches. Southern sable antelope were brought in to captivity in North America during the 1910s to 1950s (Piltz et al., 2015). In the absence of a complete pedigree since founding, we do not know which founding lines formed the current AZA populations nor the private ranch populations, and only through using genomic tools can we begin to understand some of the genetic structuring between sable antelope populations in North America and make breeding and management recommendations. The overarching aim of this study was to empirically assess how traditional zoo population management methods influence genetic diversity retention and differentiation between populations that are largely managed independently. Our two key findings are (a) reproductive planning protocols have significant impacts on population‐wide heterozygosity and inbreeding and (b) metapopulation management of public and private collections may be vital for some species to reduce genetic drift, exchange novel genetic diversity, and achieve desired population goals of demographic stability and genetic diversity retention.

### 4.1 Genetic diversity

Ex situ conservation programs aim to conserve existing genetic diversity, under the assumption that high and variable genetic diversity correlates with adaptive potential, and is key in ensuring long‐term species survival (Russello & Jensen, 2018). We found significantly higher heterozygosity and significantly lower inbreeding occurring in the AZA zoo managed populations (this includes the Fossil Rim population; a larger conservation center managed by the AZA), relative to the privately managed herds (Figure 2a,b). We propose that the different management strategies (i.e., kinship guided management versus. nonkinship guided management) are driving these findings. Similar patterns have been observed in *Peromyscus leucopus* mice, where populations managed to minimize mean kinship retain greater heterozygosity and lower inbreeding after 20 generations, compared to populations that were either randomly mating or managed for behavioral qualities (Willoughby et al., 2015). Even though we found a range of inbreeding levels across all study populations, higher inbreeding coefficients and more recent inbreeding events were observed in the private ranches (Figure 3). This is in direct comparison to the AZA population, where we observed very few occurrences of recent inbreeding (most events occurring>~8 generations past; Figure 3, AZA), suggesting that mean kinship and guided bull rotations for inbreeding avoidance are effective in reducing the occurrence of inbreeding despite the fact that the pedigree for most individuals managed in the sable antelope Species Survival Program is unknown. Fan et al. (2019) conducted similar research in forest musk deer, *Moschus berezovskii*, production farms, where males with “superior” traits are prioritized for breeding, and proposed this had created a nonrandom mating system that had increased the occurrence of inbreeding and accelerated genetic drift. However, we note that the four ranch populations of sable antelope that were genotyped represent only a very small subset of the privately owned ranch populations in North America (*N* = >3,000 individuals; Mungall, 2018), all of which have independent management strategies, and some of which may prioritize genetic‐based breeding recommendations. Genotyping complete herds, from more ranches, will allow us to better understand the genetic diversity present in the private sector.

### 4.2 Genetic structure

We found evidence of genetic differentiation and clustering between all three study populations (Figure 4). There are several nonmutually exclusive explanations for the observed genetic clustering and differentiation between our North American sable populations: (a) initial founder effects during the establishment of the North American captive populations combined with (b) several of the North American populations historically being managed in isolation may have resulted in genetic drift that has led to genetic differentiation between captive populations. Moreover, novel selection pressures experienced in captive environments may have contributed in part to the observed genetic differentiation through adaptation to captivity, and the small effective breeding size (common to most ungulate species) in captive sable antelope populations will not only accelerate potential genetic drift, but also increase the overall kinship of the entire population.

Small populations such as ex situ managed collections in zoos and conservation centers are most at risk of genetic drift, and this process is exacerbated in polygamous species that experience high reproductive skews (Gooley et al., 2018;Gustafson, Vickers, Boyce, & Ernest, 2017;Lacy, 1987;Willoughby et al., 2015). However, this is primarily managed through kinship and guided bull rotations for inbreeding avoidance, equalizing male reproductive outputs as best as possible. On the private ranches, bull rotations can and do occur, though it is not as structured as the AZA management system nor is it based on pedigree‐derived kinship values and recent pedigree history. Genetic drift occurring independently in the public and private facilities may explain the genetic clustering observed among ex situ populations of sable antelope in North America (Figure 4). These findings support the proposal of a metapopulation management system, whereby the loss of genetic diversity resulting from genetic drift can be reduced through the augmented immigration of as little as one individual per generation (Gustafson et al., 2017;Lacy, 1987).

### 4.3 Metapopulation management

In order to enable our results to be used in applied conservation management, we are working with the Source Population Alliance (SPA). The SPA is an initiative to manage multiple species as metapopulations spanning public and private sectors (https://sourcepopulation.org/). The genetic clustering observed between our North American captive populations closely emulates the artificial gene flow (or lack thereof) occurring through transfer events. For example, Fossil Rim Wildlife Center, an AZA and SPA managed population, has transferred bulls to the privately owned ranch populations studied herein, and this is reflected in our principal component analysis (Figure 4), where we see overlap between the two population clusters. In comparison, our collectively termed AZA population has mostly been managed in isolation from the privately owned ranch populations; this too is reflected in our principal component analysis (Figure 4) where we see minimal‐to‐no overlap in the two population clusters. As a proof of concept, we advised a bull from Fossil Rim to be transferred to The Wilds, which, during our study period, successfully sired two offspring, which we genotyped using our targeted sequence capture panel. The two offspring can be observed in Figure 4, located directly between our AZA cluster and Fossil Rim cluster. These individuals not only demonstrate the relatedness between offspring and parents originating from two different “sources,” but also how metapopulation management can reduce the genetic differentiation between facilities. The value of managing captive sable antelope as a metapopulation including public and private breeding facilities, as proposed by the SPA, is exemplified here. We acknowledge that only a subset of North American sable antelope were genotyped herein, and therefore, our dataset does not represent a full characterization of the genetic diversity present in all North American sable antelope. This may indeed result in either underestimating or overestimating the genetic differentiation between facilities, and thus, further data collection would be required to gain a complete understanding of how best to empirically manage this species.

Nevertheless, our results demonstrate that transfers of individuals between the different breeding facilities may act to reduce the occurrence of genetic drift in captivity, better maintain population‐wide genetic diversity and reduce the occurrence of inbreeding (Weeks, Stoklosa, & Hoffmann, 2016). Similar results have been observed in the forest musk deer, wherein of three captive populations, the highest genetic diversity was observed in the only population to exchange individuals with surrounding musk deer farms (Fan et al., 2019). As acquiring wild founders is presently not achievable for the North American sable antelope populations, we suggest that the larger managed populations (e.g., Fossil Rim) can act as gene pools for transfers to smaller and closer proximity populations within both the public and private breeding facilities. Male rotations, based on molecular mean kinship, inbreeding avoidance, and molecular relatedness, will be the most effective way to increase the effective population size and reduce the impact of genetic drift.

### 4.4 Future directions

Using the species‐specific sequence capture genotyping tool developed herein, our next goal is to integrate genomic data into metapopulation management. Creating a framework for molecular kinship‐based breeding recommendations and exchanging novel genetic diversity between the different public and private breeding facilities is a future goal and currently underway in our research team. Furthermore, we plan to compare genomic diversity of sable antelope from Africa (both wild and captive), to our North America sable antelope, as to understand how genomic diversity has differentiated since the founding of the North American ex situ populations.

## ACKNOWLEDGEMENT

The authors thank: Jill M. Piltz (Disney’s Animal Kingdom), Dr. Douglas L Armstrong (Omaha’s Henry Doorly Zoo and Aquarium), Dan Beetem (The Wilds), Kevin and Cole Reid (Stewards for Wildlife), Jimmy and Justin Gregory (Austin Savanna), Hayden Kelly (Griffin Point Ranch), and anonymously participating Conservation Centers for Species Survival’s Source Population Alliance members for providing samples. We thank Alison Devault from Arbor Biosciences for the construction and quality checking of the sable antelope myBaits array and for comments on the manuscript. We also thank Dr. Janine Brown, Smithsonian Conservation Biology Institute for substantial discussions during early stages of this project and funding support and Ms. Jill Piltz, Co‐Ordinator ‐ AZA Sable Antelope Species Survival Plan for regular feedback and guidance throughout this project. K.P.K. and the research reported herein was supported by the Smithsonian Institution Competitive Grants Program, the Sichel Endowment, and the Phil Reed Fund. G.T. was supported in part by funding provided through the Smithsonian Institution’s Short‐Term Visitor Fellowship program.

## CONFLICT OF INTEREST

None declared.

## AUTHOR CONTRIBUTIONS

J.E.M, B.S.P. D.E.W. and K.P.K designed the study; H.H. coordinated sample collection from Fossil Rim; B.S.P. coordinated the collection and curation of all samples; S.C.R. and K.R.M. performed DNA extractions, library preparation and capture enrichment; G.T., P.D. and K.P.K. designed the sable antelope targeted sequence capture array; R.M.G., G.T., P.D., B.S.P., C.W.E. and K.P.K. planned the analysis approach; R.M.G. performed variant calling and population genetic analyses; G.T. visualized the distribution of SNPs across the sable antelope genome; G.F. provided management and transfer information for captive sable antelope and guidance for research implementation. R.M.G. drafted the manuscript and all authors revised the manuscript and approved and contributed to the final version.

## Supporting information

Fig S1

Fig S2

## Data Availability

Unfiltered.vcf files, sample ID information, and R codes used for analyses are available from the following data repository: https://figshare.com/projects/Comparison_of_genomic_diversity_and_structure_of_sable_antelope_Hippotragus_niger_in_zoos_breeding_centers_and_private_ranches_in_North_America/74874
