# Supplementary figures and images for: Comparison of genomic diversity and structure of sable antelope (Hippotragus niger) in zoos, conservation centers, and private ranches in North America
Source: Evol Appl. 2020 Apr 27;13(8):2143–54. doi: 10.1111/eva.12976 (PMC7463370; doi:10.1111/eva.12976)

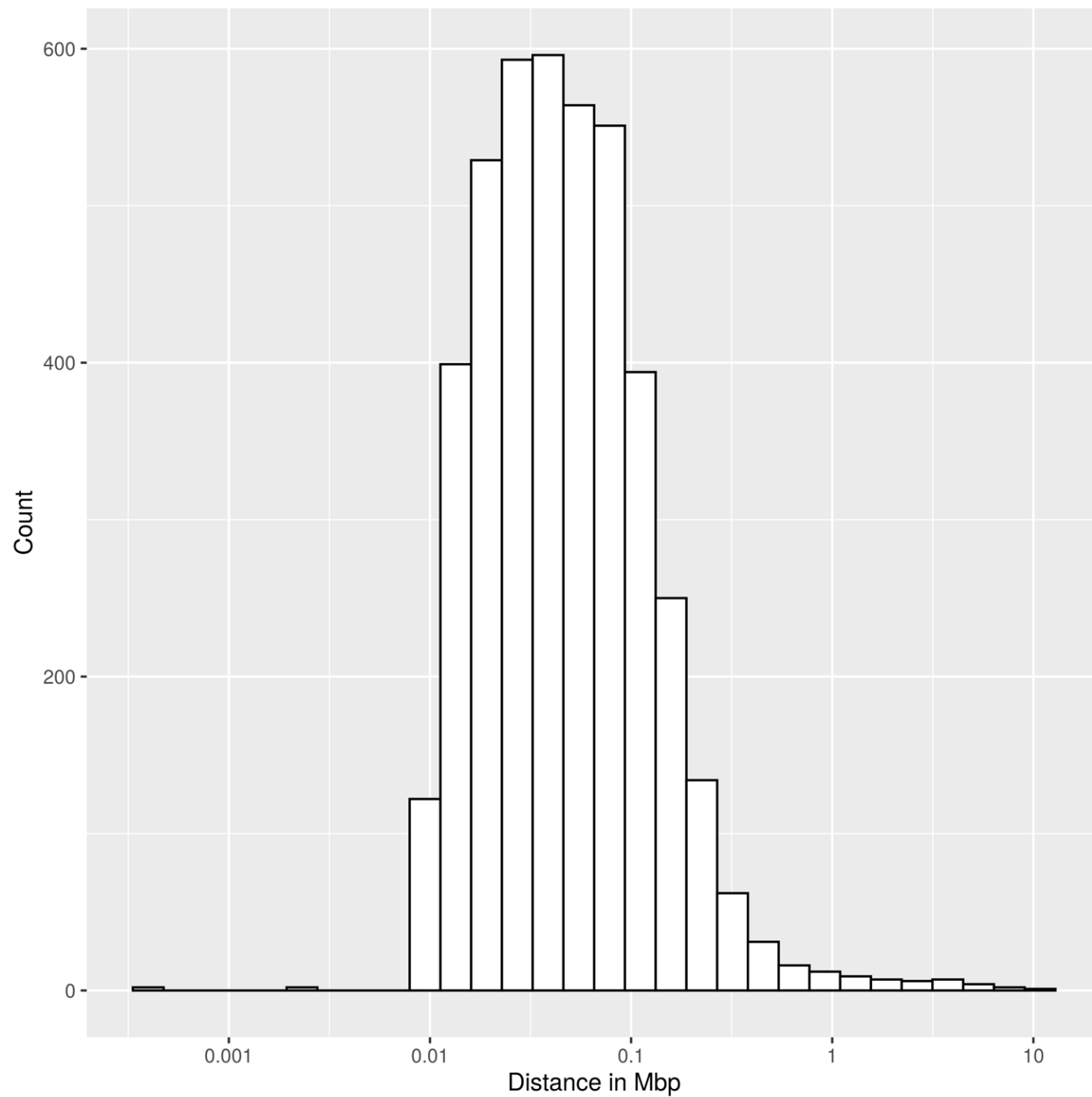

Supplement: Supplementary file 1 — Fig S1 [file EVA-13-2143-s001.pdf]

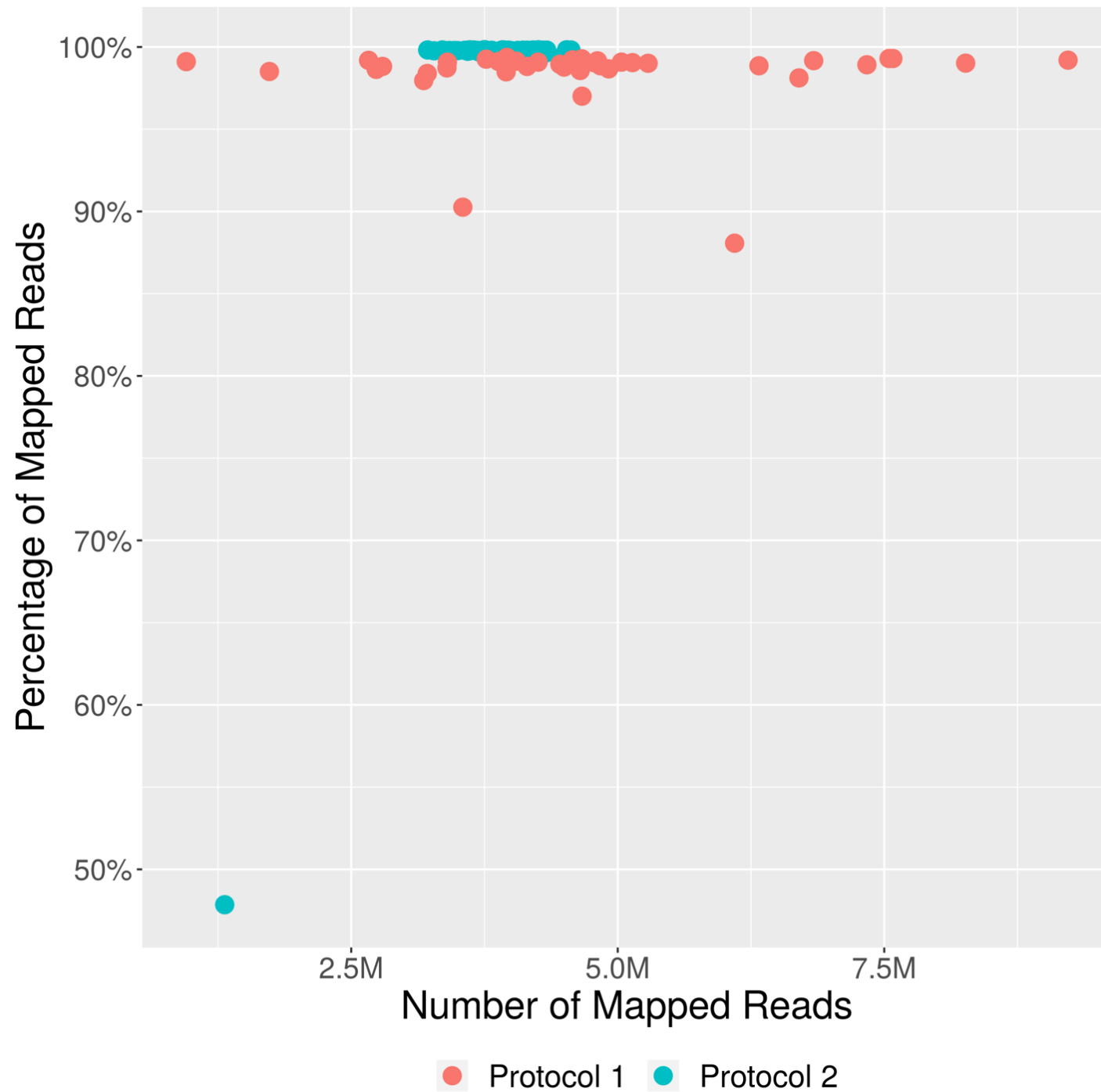

Supplement: Supplementary file 2 — Fig S2 [file EVA-13-2143-s002.pdf]
